# OntologyWidget – a reusable, embeddable widget for easily locating ontology terms

**DOI:** 10.1186/1471-2105-8-338

**Published:** 2007-09-13

**Authors:** Catherine C Beauheim, Farrell Wymore, Michael Nitzberg, Zachariah K Zachariah, Heng Jin, JH Pate Skene, Catherine A Ball, Gavin Sherlock

**Affiliations:** 1 Department of Genetics, Stanford University School of Medicine, Stanford, CA 94305-5120, USA; 2 Department of Biochemistry, Stanford University School of Medicine, Stanford, CA 94305-5307, USA; 3 Department of Neurobiology, Duke University Medical Center, Durham, NC 27710, USA

## Abstract

**Background:**

Biomedical ontologies are being widely used to annotate biological data in a computer-accessible, consistent and well-defined manner. However, due to their size and complexity, annotating data with appropriate terms from an ontology is often challenging for experts and non-experts alike, because there exist few tools that allow one to quickly find relevant ontology terms to easily populate a web form.

**Results:**

We have produced a tool, OntologyWidget, which allows users to rapidly search for and browse ontology terms. OntologyWidget can easily be embedded in other web-based applications. OntologyWidget is written using AJAX (Asynchronous JavaScript and XML) and has two related elements. The first is a dynamic auto-complete ontology search feature. As a user enters characters into the search box, the appropriate ontology is queried remotely for terms that match the typed-in text, and the query results populate a drop-down list with all potential matches. Upon selection of a term from the list, the user can locate this term within a generic and dynamic ontology browser, which comprises the second element of the tool. The ontology browser shows the paths from a selected term to the root as well as parent/child tree hierarchies. We have implemented web services at the Stanford Microarray Database (SMD), which provide the OntologyWidget with access to over 40 ontologies from the Open Biological Ontology (OBO) website [1]. Each ontology is updated weekly. Adopters of the OntologyWidget can either use SMD's web services, or elect to rely on their own. Deploying the OntologyWidget can be accomplished in three simple steps: (1) install Apache Tomcat [2] on one's web server, (2) download and install the OntologyWidget servlet stub that provides access to the SMD ontology web services, and (3) create an html (HyperText Markup Language) file that refers to the OntologyWidget using a simple, well-defined format.

**Conclusion:**

We have developed OntologyWidget, an easy-to-use ontology search and display tool that can be used on any web page by creating a simple html description. OntologyWidget provides a rapid auto-complete search function paired with an interactive tree display. We have developed a web service layer that communicates between the web page interface and a database of ontology terms. We currently store 40 of the ontologies from the OBO website [1], as well as a several others. These ontologies are automatically updated on a weekly basis. OntologyWidget can be used in any web-based application to take advantage of the ontologies we provide via web services or any other ontology that is provided elsewhere in the correct format. The full source code for the JavaScript and description of the OntologyWidget is available from .

## Background

Although there are many potential applications, we developed OntologyWidget to help microarray researchers use ontologies and controlled vocabularies to describe and annotate their experiments. Using biomedical ontologies to annotate experimental designs, experimental variables and biological samples helps to achieve consistency, to provide the opportunity for complex searches and to allow computational access to the annotations. However, in the process of annotating a single investigation, a researcher might require several large and complex ontologies. For example, the NCBI Taxonomy might be used to describe the organism used as the source of the biological sample being studied, while an anatomy ontology might be used to describe the tissue that was sampled. Because most experimental annotations are best provided by the researcher(s) who conducted the experiments, and it is unlikely that bench biologists would have had the time to become power users of several ontologies, we wanted to provide an easier method to use ontologies that would be rapid and require no expert knowledge of the ontology. Thus, in an attempt to ease the experimental annotation process and decrease the burden of entering high-quality annotations, we developed the OntologyWidget. OntologyWidget fulfilled our requirements to (1) provide a graphical, web-based tool, (2) have a fast auto-complete term search, (3) enable users to view and browse ontology structures, (4) provide a simple means to search more than one ontology, (5) provide the means of collecting ontology terms to post to another web page or to another program and (6) be deployed by our collaborators and others with a minimum of effort.

As of August 2007, there were 66 ontologies listed on the Open Biomedical Ontologies web site [1], describing biological concepts that range from development to anatomy to taxonomy to experimental approaches. Several tools already exist to search and browse ontologies, such as the Amigo Browser and the Ontology Lookup Service (OLS) from the EBI. The Amigo browser [3] is a web-based tool that allows a user to browse, query and visualize terms from any ontology formatted in the OBO format. It can be installed as a Perl Application, provides a normal text search as well as a convenient means to browse and explore trees. However, Amigo does not allow one to select terms that can then be posted to another web form, its installation is fairly complex, and it does not allow users to dynamically find terms within the ontology while typing. The Ontology Lookup Service [4] from the EBI, which was published while this work was in progress, supports many of the same ontologies that we do and has similar browse and auto-complete features. The OLS also identifies the relationship type between terms, gives associated information and descriptions for the terms where available. It fulfills many of our needs, except that is part of a much larger software package. Its installation has a large footprint requiring around 40 Java jar files. Therefore, the OLS did not fulfill one of our requirements, that it is easy for our collaborators to install and use on their websites. Thus, we developed the OntologyWidget, which can be embedded in a web page in a relatively straightforward fashion, and used with other web-based annotation applications.

## Results and discussion

### Features of OntologyWidget

**Figure 1:**
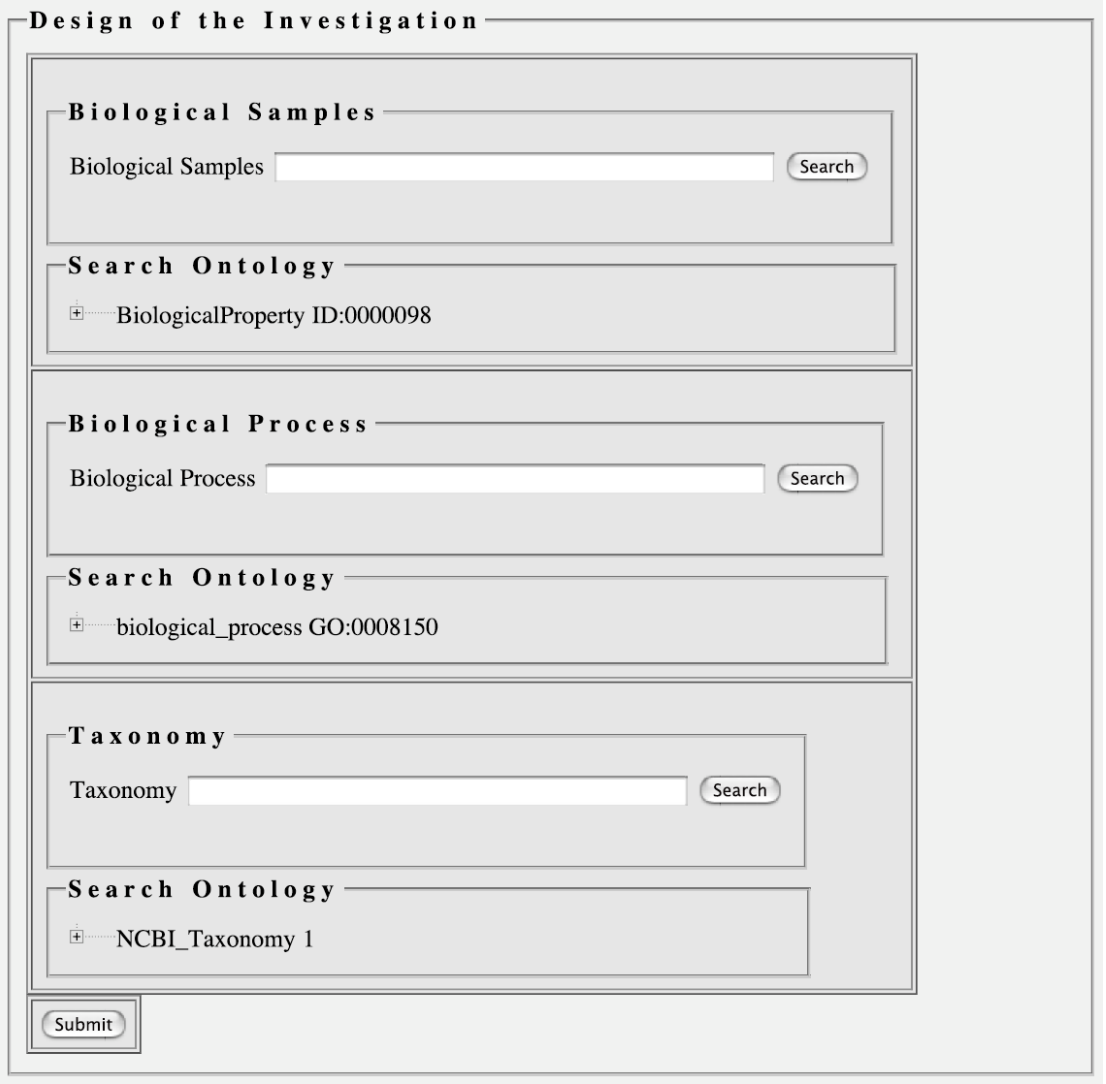
**Ontology widgets on an html page**. Three instances of the OntologyWidget are shown on a single html page. Each widget contains both an autocomplete box, into which text can be typed, and an ontology browser, which can either be browsed from the top level, or can be opened at a selected node, based on the entered term. Each instance of the widget is connected to a different ontology.

An instance of the OntologyWidget in a web page can be used to search and browse a single ontology, but multiple instances of the OntologyWidget can also be embedded in a single web page, allowing a user to rapidly annotate a biological entity with terms from several ontologies using the same web page. In addition, the OntologyWidget can be configured to use only a specific subsection of an ontology, by specifying a particular node to be used as a root. In the example provided in Figure 1, three different ontologies are presented to the user: part of the MGED Ontology [5], the Biological Process portion of the Gene Ontology [6] and the NCBI taxonomy [7]. In Figure 1, both the MGED Ontology (MO) and the Gene Ontology are shown with different terms presented as the root terms. In the case of the MGED Ontology, we have limited the widget to use "BiologicalProperty" as the root term. Thus, the top widget could be used to annotate one aspect of the experimental approach that was used, as this subgraph of MO lists various experimental designs. Limiting terms provides accuracy and ease of use – users will not accidentally enter terms that are incorrect for a given context. We can also specify a default selection for the ontology terms in the cases where a single term is commonly used in many situations (for example, using "*Homo sapiens*" as the default term when most of the research being conducted is based on human samples. As of this writing, SMD Ontology Web Services provide access to over 40 ontologies from the OBO site [1] for use with the OntologyWidget, though we have plans to incorporate all the ontologies on the OBO website.

**Figure 2:**
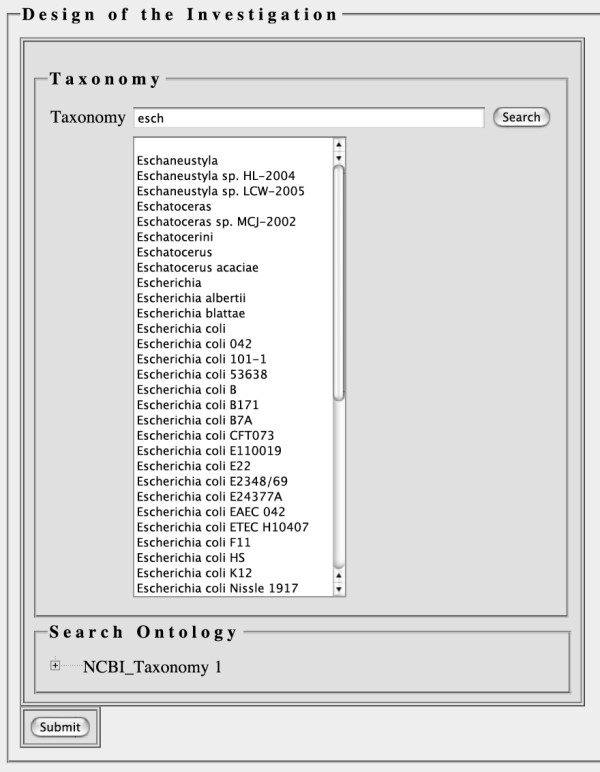
**Auto-complete functionality**. A user can begin typing part of a term (including wild-card characters), and potential completions of the typed in phrase are displayed in the drop down menu. In this case, potential completions of 'esch' from the NCBI taxonomy are shown.

Sometimes, a user of an ontology merely needs to find the correct term and does not need to know the context of an ontology term. In this case, OntologyWidget's text search and auto complete functions will be sufficient. The screenshot provided in Figure 2 illustrates OntologyWidget's auto-complete function in an example using the NCBI taxonomy. Note that the letters "esch" were typed into the text box and a long list of taxonomic terms that start with those letters are available for selection. The OntologyWidget text search also accepts the wildcard character, *, and is case-insensitive. When a user identifies the correct term in the list of results, it can be selected to populate the text box. We limit the number of matching terms to 50 for the sake of performance. The last item on the list is "More terms available ..." in this case to indicate that more than 50 terms match the current typed-in sequence of characters. The user is required to refine their search by typing in more characters to see a more specific subset of the terms.

**Figure 3:**
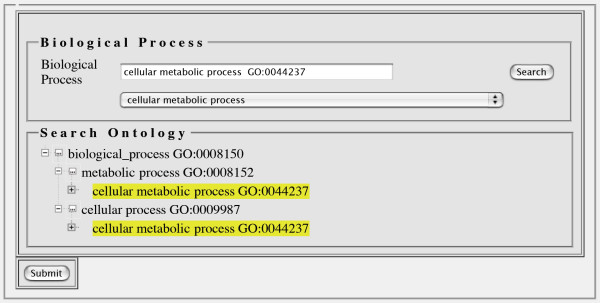
**The ontology browser display**. When an autocompleted term is selected and the 'Search' button is clicked, all the paths from the selected term ("cellular metabolic process") to the root of the ontology ("Biological Process") are shown. Instances of the search term are highlighted as a visual aid. Selection of a more or less specific term from the ontology browser will populate the text box with that term.

At other times, it is important for a user to explore the context of an ontology term to determine if a different term (for example, one that is more specific or one that is less specific) would be more accurate for the given annotation. Figure 3 shows a screenshot of the ontology tree browser being displayed after a user entered a term into the text box and then clicked on the "Search" button. In this case, the term "cellular metabolic process" is highlighted among a nested set of parent terms all the way up to the Gene Ontology's Biological Process term. In this ontology browser display, children of a given node can either be revealed using the "+" icon or hidden using the "-" icon. Note that while the ontology tree browser display shows the direct path (or paths) from the root term to the input term, other child terms of other nodes within those paths are not displayed. Instead, a "..." icon is used to indicate that additional children exist for that parent node. When the user clicks the "..." icon, all the immediate child terms are displayed and the "..." icon disappears. The use of the "..." icon allows the initial ontology tree browser display to remain relatively uncluttered, and easier to understand and navigate. At this time, the widget does not display the details or description of an ontology term. We maintain this information in our database, and it is provided via our web services API, so future updates to our software will make it possible to dynamically provide this information.

OntologyWidget has been tested on both MacOSX and Windows XP, and is known to run successfully in Safari 2.0.4, Firefox 1.5.12, Firefox 2.0.0.4, Opera 9.02, and Mozilla 1.7.13 on MacOSX, and Internet Explorer 6 and 7, Firefox 1.5.8, and Opera 9.1 on Windows XP.

### Implementation

**Figure 4:**
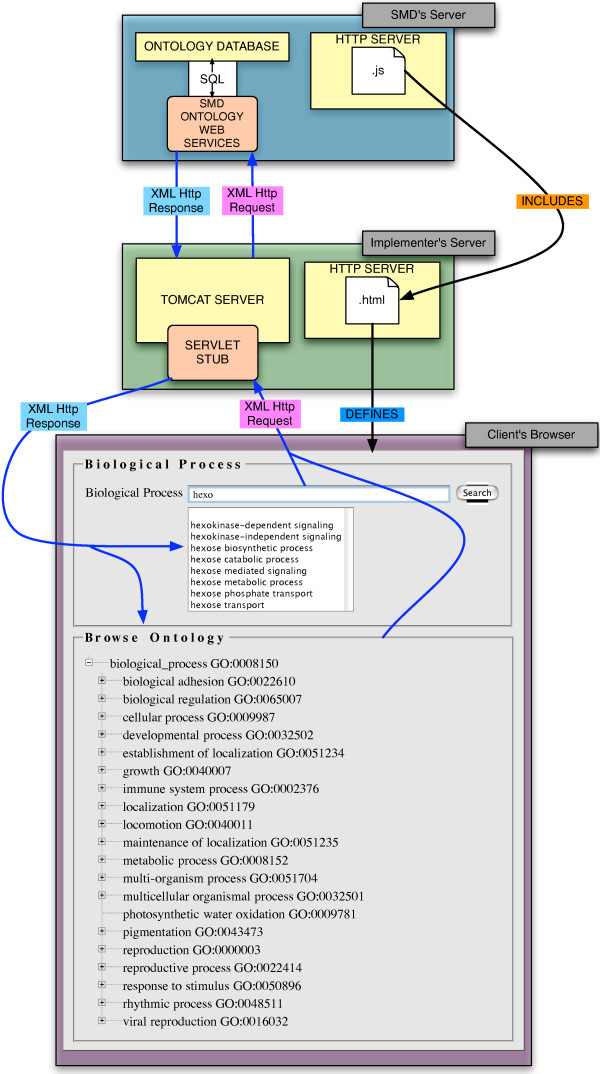
**System Architecture of the OntologyWidget**. Queries from the two components of the OntologyWidget are sent to the SMD Ontology Web service (either directly, in the case of the SMD deployment, or indirectly via the servlet stub in the case of a third party deployment), and are answered with an XML Http Response, sent to the client's browser, in the case shown, via the servlet stub. The JavaScript running in the client's browser then renders the XML, either into the Ontology Browser, or by indicating potential autocomplete terms.

The user-interface of the OntologyWidget is written in JavaScript and html with its dynamic content delivered with AJAX (Asynchronous JavaScript and XML) technology. On the server side, Java servlets receive the user requests, submit queries to a database, and return XML to the JavaScript interface. In order to provide access to the Widget across internet domains and to circumvent cross-site scripting issues, either a client-side servlet stub (in the form of the .war file that is provided in the OntologyWidget download) must be installed on the implementer's web server, or a reverse proxy web server must be created (both of which are explicitly detailed in the documentation that accompanies the OntologyWidget download). Although the JavaScript files on the SMD site that provide the OntologyWidget user interface can simply be included via http in the html of the implementing web page, they are also available for download and local deployment through our web site [8]. The .war file for deploying the proxy servlet, documentation, and a sample html file are also available at the same web site. Figure 4 depicts the overall system architecture, showing how the JavaScript and ontology web services residing on our system can be used by web pages hosted elsewhere.

Once the implementing web server has Apache Tomcat [2] successfully installed and operational, the second step to use OntologyWidget is to install the OntologyWidget Tomcat Servlet stub provided in the .war file. The Tomcat Servlet stub provides computers outside of our domain with access to the SMD Ontology Server. This enables users to create their own html pages that will call our JavaScript program, which communicate with the Ontology Server. The Servlet stub should be installed in the client's Tomcat instance in the *webapps *directory.

### Client user interface via html

To incorporate the OntologyWidget on one's own web page, an html file must be created that correctly implements the OntologyWidget. Detailed instructions for incorporating the OntologyWidget into web pages are provided in the help documentation at our website, but here we briefly describe some of the necessary steps, and their purposes. First, the html file must initialize the *SMDService *module (described below in more detail). Second, the html file must define OntologyWidget parameters, and third, the html file must provide page locations for each OntologyWidget used on the page, and provide the hidden fields which report the data selected upon submission. Lastly, the URL for the location of the servlet proxy must be defined.

In the *<head> *section of the html file, the seven JavaScript files, which construct the OntologyWidget plus icon image files and a cascading style sheet, are defined by the reference to each JavaScript file in lines beginning *<link> *and *<script >*. The tree in the annotation widget is built using YAHOO's public domain user interface code for widgets [9].

The second part of the *<head> *section contains five JavaScript functions that define the widgets, start the *SMDService *module, and manage the query threads. When the page loads, the *window.onload *function, initiates an interval checker that manages query threads, and starts the *SMDService *module that makes a connection to the SMD Ontology web services.

An instance of an OntologyWidget simply requires six pieces of data to be initialized:

• title that will be displayed on the OntologyWidget (string)

• ontology ID (database identifier, supplied by function call)

• ontology name (string)

• term name (string)

• label (string)

• unique identifier for the instance of the OntologyWidget in the html file (string or number)

The title and label provide context both to the user and to the author of the html file. Because one can put multiple OntologyWidgets on a single web page, the unique identifier (uid) is needed to keep queries and data directed to the correct instance of the OntologyWidget.

In the body of the html file, the form and the URL of the widget's action function are defined. Within the html form element, the location of each OntologyWidget instance is defined. When the page is posted, that is when a post or submit button is clicked, these data associated with each widget instance (and any other fields on the html page that the page implementer has chosen to be posted) will be submitted via an implementer-written submit function. As an illustration, the example html file included in the OntologyWidget download contains an example submit function that simply reports these fields in a table.

### SMD Ontology Server

The SMD Ontology Server comprises a package of Java classes that receive http requests, query a database that contains the supported ontologies, format the data into XML and return the XML to the interface. The simplest way to use the service is through the proxy servlet that we supply as a .war file. Using this simple method, the proxy servlet is installed within a Tomcat server running on the implementer's server and the location of the servlet is referenced in all html pages that contain an instance of the OntologyWidget. Our Web Serviceapplication programming interfaces (APIs) are available, should the implementer wish to create their own software to use our web services. While we do not allow unrestricted programmatic access to our implementation because our database houses a lot of other data, the code can be obtained in the SMD code release.

The SMD Ontology Service is implemented as a servlet and responds to eight requests: *getOntologyList*, *getChildren*, *getParents*, *getTermsByName*, *getRootTerms*, *getTerm*, *getAncestors *and *getDescendents*. A complete API description is available on our website. Each request is processed by parsing the arguments, performing the database query necessary to fulfill the function, converting the query result into an XML string, and returning this string as the reply to the request.

Since performance is a paramount issue for this application, the Ontology Service diverges from typical web services in that SOAP marshalling and unmarshalling software are not used. These systems, while generally applicable, typically incur a large amount of overhead and a performance cost. We discovered that our ontology schemas that flattened the data caused unreasonable performance response for the type-in suggestion box. The performance hit was particularly evident when we were displaying a partial ontology. All terms that were suggested matches had to fall within the subtree of the selected root term. To address this problem, we have implemented a data caching scheme for the SMD Ontology Service on the server side. We created a data structure that holds all the paths from an ontology's root term to all its child terms. We serialized the path data structure into a Binary Large Object (BLOB). When Tomcat starts, a context listener class is notified that reads the BLOBs from the database and holds the data in memory for rapid access. Thus, some queries to the SMD Ontology Service do not need to query the database directly, resulting in a significant performance improvement.

## Conclusion

OntologyWidget is an easy-to-use ontology search and display tool that can be used on any web page. Implementers will be able to take advantage of over 40 ontologies provided by our SMD Ontology Services and will not have to store or update their own copies of ontology data. Because implementers using OntologyWidget can simply include the JavaScript stored on our system, they will be able to benefit from bug fixes and incremental enhancements that we deploy, without having to download and install updated software.

OntologyWidget has already been implemented by collaborators maintaining the NIH Neuroscience Microarray Consortium website [10]. As implemented there, OntologyWidget is used to help researchers describe the anatomical regions used to generate samples from mammals.

## Availability and requirements

Project name: The Ontology Widget;

Project home page: ;

Operating system: Platform independent;

Programming language: JavaScript, Java, html; Other requirements: Tomcat 4.0 or higher;

License: MIT Open Source License;

A web page that demonstrates the OntologyWidget can be accessed from the project's home page [8]. This example page allows users to use OntologyWidget to explore a small subset of the MGED Ontology, the biological_process section of the Gene Ontology and the entire NCBI taxonomy. Documentation about the software and the war file that needs to be installed can be accessed by visiting the project home page [8]. Although it is not necessary to download or install the JavaScript source files, they are also available from the project website [8]. OntologyWidget uses the MIT Open Source license that permits anyone to use, re-use, edit or redistribute the software for any purpose, as long as attribution is given to the authors.

## Authors' contributions

CCB implemented the OntologyWidget, wrote the documentation, provided the example files and contributed to the writing of the manuscript. FW implemented the SMD Ontology Service, created the .war file and helped troubleshoot throughout the project. MN and ZKZ worked on the server side caching and optimization of the OntologyWidget performance. HJ created software that enters ontologies from the OBO site into SMD. JHPS provided use cases and evaluated several versions of the developing software. CAB and GS created the software specifications, created the testing document, tested software throughout the project and contributed to the writing of the manuscript and help document. All authors read and approved the final manuscript.
